# Efficacy of Ebselen Against Invasive Aspergillosis in a Murine Model

**DOI:** 10.3389/fcimb.2021.684525

**Published:** 2021-06-23

**Authors:** Karina Mayumi Sakita, Isis Regina Grenier Capoci, Pollyanna Cristina Vincenzi Conrado, Franciele Abigail Vilugron Rodrigues-Vendramini, Daniella Renata Faria, Glaucia Sayuri Arita, Tânia Cristina Alexandrino Becker, Patricia de Souza Bonfim-Mendonça, Terezinha Inez Estivalet Svidzinski, Erika Seki Kioshima

**Affiliations:** ^1^ Laboratory of Medical Mycology, Department of Clinical Analysis and Biomedicine, State University of Maringá, Maringá, Brazil; ^2^ Laboratory of General Pathology, Department of Basic Health Sciences, State University of Maringá, Maringá, Brazil

**Keywords:** ebselen, *Aspergillus*, murine model, antifungal, systemic infection

## Abstract

Invasive aspergillosis is one of the major causes of morbidity and mortality among invasive fungal infections. The search for new antifungal drugs becomes imperative when existing drugs are not able to efficiently treat these infections. Ebselen, is an organoselenium compound, already successfully approved in clinical trials as a repositioned drug for the treatment of bipolar disorder and prevention of noise-induced hearing loss. In this study, we aimed to reposition ebselen for the treatment of invasive aspergillosis by showing ebselen effectiveness in a murine model. For this, BALB/c mice were immunosuppressed and infected systemically with *Aspergillus fumigatus*. Animals were divided and treated with ebselen, voriconazole, or drug-free control, for four days. The kidneys were used for CFU count and, histopathological and cytokine analysis. Ebselen was able to significantly reduce the fungal burden in the kidneys of infected mice with efficacy comparable with voriconazole treatment as both had reductions to the same extent. The absence of hyphae and intact kidney tissue structure observed in the histopathological sections analyzed from treated groups corroborate with the downregulation of IL-6 and TNF. In summary, this study brings for the first time *in vivo* evidence of ebselen efficacy against invasive aspergillosis. Despite these promising results, more animal studies are warranted to evaluate the potential role of ebselen as an alternative option for the management of invasive aspergillosis in humans.

## Introduction

Invasive aspergillosis (IA) remains one of the major causes of morbidity and mortality among invasive fungal infections, especially in intensive care unit patients (Ostrosky-Zeichner and Al-Obaidi, 2017). Voriconazole is the gold standard for treatment of IA (Patterson et al., 2016). Despite this, the mortality in patients who received appropriate initial voriconazole therapy is up to 24% (Lestrade et al., 2019). Patients with invasive aspergillosis caused by azole-resistant *A. fumigatus* showed 100% all-cause mortality at 100 days (Cho et al., 2019).

Facing this critical scenario, in an attempt to optimize the process of searching for new drug options, repositioning drugs has become an interesting approach to speed up the discovery of new antifungal drugs. This approach decreases the conventional time of drug discovery from 10–17 to 3–12 years for repurposed compounds, as it bypasses much of the discovery and preclinical stages and phase I studies of safety (Farha and Brown, 2019).

Ebselen [2-phenyl-1,2-benzoselenazol-3(2H)-one; EbSe] is an organoselenium compound already successfully tested in human clinical trials for neuroprotective effect (Saito et al., 1998), treatment for bipolar disorder (Masaki et al., 2016), and prevention of noise-induced hearing loss (Kil et al., 2017) with no reported side effects or toxicity. Recently it also has been approved for clinical trials on moderate COVID-19 patients (NCT04484025) (ClinicalTrials.gov, 2020). In addition, the antifungal *in vitro* activity of EbSe against *Candida* spp., *Trichosporon asahii*, and *Cryptococcus* spp. has been demonstrated (Thangamani et al., 2017; Kubiça et al., 2019). Recently, Marshall and colleagues (2019) proved the ability of EbSe to block *A. fumigatus* thioredoxin reductase (TrxR) activity (Marshall et al., 2019). This flavoenzyme has been largely studied by our group as a promising target for antifungal drugs (Capoci et al., 2019; Rodrigues-Vendramini et al., 2019; Faria et al., 2020). In fact, differences between TrxR from humans and fungi lead EbSe to exert different effects in fungi TrxR by accumulation of reactive oxygen species (ROS) and cell death (Ren et al., 2018). Recently, Binder et al. (2020) showed that the *trxR* gene is essential for *A. fumigatus* survival and has only 28% of homology to its human ortholog.

To the best of our knowledge, this is the first study demonstrating the efficacy of ebselen antifungal treatment *in vivo*. Thus, the aim of this study was to bring evidence of EbSe effectiveness in invasive aspergillosis using a murine model.

## Materials and Methods

### Antifungal Agents

The following compounds were used for susceptibility tests: ebselen (EbSe; C_13_H_9_NOSe; TargetMol), voriconazole (VOR; Pfizer Incorporated, New York, NY, USA), and amphotericin B (AMB; Sigma-Aldrich, St. Louis, MO, USA). Stock solution of voriconazole was prepared in Dimethyl Sulfoxide (DMSO; Sigma-Aldrich, St. Louis, MO, USA). DMSO and Pluronic^®^ F-127 (Sigma-Aldrich, St. Louis, MO, USA) were used for the solubilization of ebselen. For *in vivo* treatment, we used voriconazole injectable solution (VOR; Cristalia Prod. Quim. Farm. Ltda., Itapira, SP, Brazil) diluted in phosphate saline buffer (PBS) and the ebselen stock solution (50 mg.ml^−1^ in DMSO) prepared in PBS with Pluronic^®^ F-127 (1.25%). The control group was treated with vehicle (PBS, DMSO, and 1.25% Pluronic^®^ F-127).

### Organisms and Inoculum Preparation

*Aspergillus fumigatus* reference strain (ATCC 64026) and two clinical isolates of *A. fumigatus* isolated from sputum and bronchoalveolar lavage (Af1 and Af2) were used. The collection of isolates was carried out in accordance with the regulations of the *Comitê de Ética em Pesquisa Envolvendo Seres Humanos* of the *Universidade Estadual de Maringá*, Brazil (Approval n° 2.748.843). The sample collection was performed by healthcare professionals at the *Hospital Universitário de Maringá* (HU) and at the *Laboratório de Ensino e Pesquisa em Análises Clínicas* (LEPAC). For inoculum preparation, the strains were grown on potato dextrose agar (PDA) at 35°C for 7 days. Conidia were harvested with 0.1% Tween 80 in saline (0.85%). Homogenous conidial suspensions were collected following filtration through a sterile syringe with cotton and then adjusted to the desired concentration.

### Minimum Inhibitory Concentration Determination

The procedures were performed according to the broth microdilution protocol from the clinical & laboratory standards institute (CLSI) M38-A2. For the interpretation of results, 0.02% of resazurin sodium salt (C_12_H_6_NNaO_4_; R7017, Sigma, St. Louis, MO) was added after 24 h and incubated for an additional 24 h at 35°C. A blue color was interpreted as the absence of metabolic activity (no spore germination). A fluorescent pink color was interpreted as the presence of metabolic activity (spore germination), and a purple color was interpreted as a trailing result, which means that some metabolic activity was present and a longer incubation time would allow the purple color to change to pink.

### Experimental Model of Invasive Aspergillosis *In Vivo*

The procedures were carried out in accordance with the regulations of the Institutional Ethics Committee for animal experimentation of the State University of Maringá, Brazil (Approval n° CEUA 9067030518). A total of 21 female BALB/c mice, weighing 22–25 g were used. Animals were housed in filter top cages and allowed access to food and water *ad libitum*. To induce an immunosuppressed state, intraperitoneal injections of cyclophosphamide (200 mg.kg^−1^ on day −3, on day 0 (day of infection), and every 3 days until the end of the experiment) were applied. Animals were infected with 1–2 × 10^4^ conidia of *A. fumigatus* (strain ATCC 64026) suspended in 100 µl of saline (0.85%) by lateral tail vein injection and were left for 24 h before starting the treatment.

The infected mice (n = 21) were randomly divided into three experimental groups: Ebselen (seven mice treated with 10 mg.kg^−1^/765.8 µmoles per mouse of ebselen), voriconazole (seven mice treated with 10 mg.kg^−1^/572.5 µmoles per mouse of voriconazole), and control (seven mice treated with solubilization buffer used as a placebo). All treatments were intraperitoneally administered, twice daily for four days. On day 5 post-infection, animals were anesthetized with isoflurane (Isoforine^®^, Cristália, SP, BR), and blood samples were collected in microtubes and centrifuged (5,000 rpm for 5 min). The serum was then stored at −80°C for cytokine measurement. After that, the animals were euthanized, and the right kidneys were aseptically removed, weighed, and mechanically homogenized in sterile saline (0.85%). Serial 10-fold dilutions of the homogenates in saline were placed on PDA and incubated for 48 h at 35°C to quantify the fungal burden in the kidneys measured as log_10_ CFU per gram of tissue. The kidney homogenates were centrifuged (11,000 rpm for 13 min), and tissue supernatants were collected and stored at −80°C for cytokine measurement.

### Histopathological Analysis

For histopathological evaluations, the left kidneys of all animals were collected, immediately fixed in 4% paraformaldehyde, paraffin-embedded, and cut into thin sections (5 μm). The sections were stained by Grocott–Gomori’s methenamine silver (GMS) to visualize fungi and counterstained with hematoxylin and eosin (H&E) for characterization of host cells. Slides were observed and photographed using a binocular light microscope (Motic BA310) with a camera (Moticam 5) coupled to a computer using Motic Images Plus 2.0 software.

### Cytokines

Cytokines in serum samples and kidney homogenate supernatants of five animals per group were measured with a BD™ Cytometric Bead Array (CBA) Mouse Inflammation Kit (BD Bioscience, San Jose, CA, USA). The kit was used for the simultaneous detection of mouse interleukin-6 (IL-6), interleukin-10 (IL-10), monocyte chemoattractant protein-1 (MCP-1), interferon-*γ* (IFN-*γ*), tumor necrosis factor (TNF), and interleukin-12p70 (IL-12p70) in a single sample following the manufacturer’s protocol. Samples were measured on the BD FACSCalibur Flow Cytometer and analyzed by FCAP Array™ Software Version 3.0 (BD Bioscience).

### Statistical Analysis

The statistical significance of the differences observed between mice treated with placebo and EbSe or voriconazole was analyzed by applying an unpaired t-test using the GraphPad Prism 5 software package (GraphPad Software Inc., San Diego, CA, USA). *P <*0.05 was considered significant in these analyses.

## Results

### Antifungal Susceptibility Testing

In general, all strains tested showed the same susceptibility profile standard *in vitro* (**Table 1**). *A. fumigatus* reference strain and Af1 and Af2 clinical isolates showed the same MIC values for EbSe and voriconazole: 4.0 µg.ml^−1^ (14.6 µM) and 0.25 µg.ml^−1^ (0.27 µM), respectively.

**Table 1:** Susceptibility profile of *A. fumigatus* reference and clinical strains against ebselen and standard antifungals.

*A. fumigatus* Strains	MIC (µg.ml^−1^/µM)		
	Ebselen (0.5–256/1.82–933.6)	Amphotericin B (0.03–16/0.03–17.3)	Voriconazole (0.03–16/0.08–45.8)
ATCC 64026	4.0/14.6	0.25/0.27	0.25/0.71
Af1	4.0/14.6	0.25/0.27	0.25/0.71
Af2	4.0/14.6	0.12/0.13	0.25/0.71

Minimum inhibitory concentration; Af1, A. fumigatus clinical isolate 1; Af2, A. fumigatus clinical isolate 2.

### Ebselen Was Able to Significantly Reduce the Fungal Burden in a Model of Invasive Aspergillosis *In Vivo*

The immunosuppressed condition of each mouse was monitored by counting the polymorphonuclear cells from the blood on days −3, 0, and +4 post-infection (d.p.i.). All animals were immunosuppressed on day 0 and continued in this condition until day +4 (data not shown).

**Figure 1:**
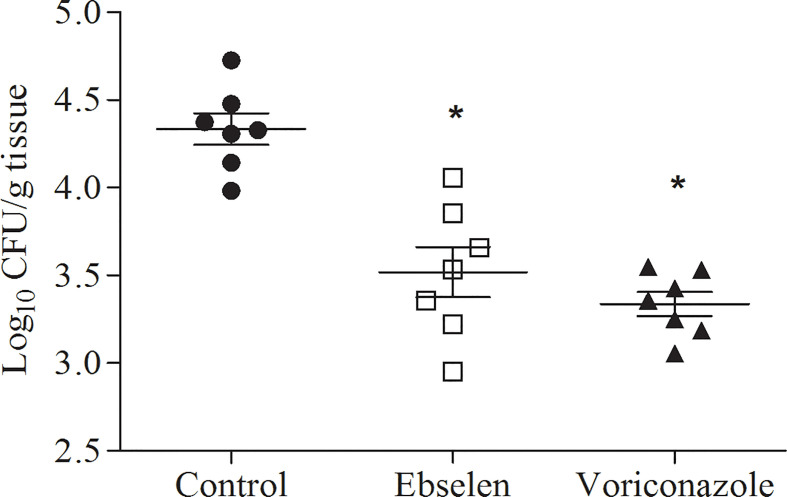
Fungal burden in the kidney after systemic infection by *A. fumigatus* (ATCC 64026). Control: mice treated with placebo; ebselen: mice treated with 10 mg.kg^–1^ (765.8 μmoles per mouse) of ebselen; voriconazole: mice treated with 10 mg.kg^–1^ (572.5 µmoles per mouse) of voriconazole. All groups were treated intraperitoneally twice daily for 4 days starting 1 day after infection. *p < 0.05. Error bars correspond to the standard deviation.

The amount of CFUs recovered after 4 days post-infection from the kidneys of mice treated with ebselen was similar to that recovered from the kidneys of mice treated with voriconazole (*p* > 0.05) and significantly lower than that recovered from the kidneys of mice treated with placebo (*p* < 0.05) (**Figure 1**).

**Figure 2:**
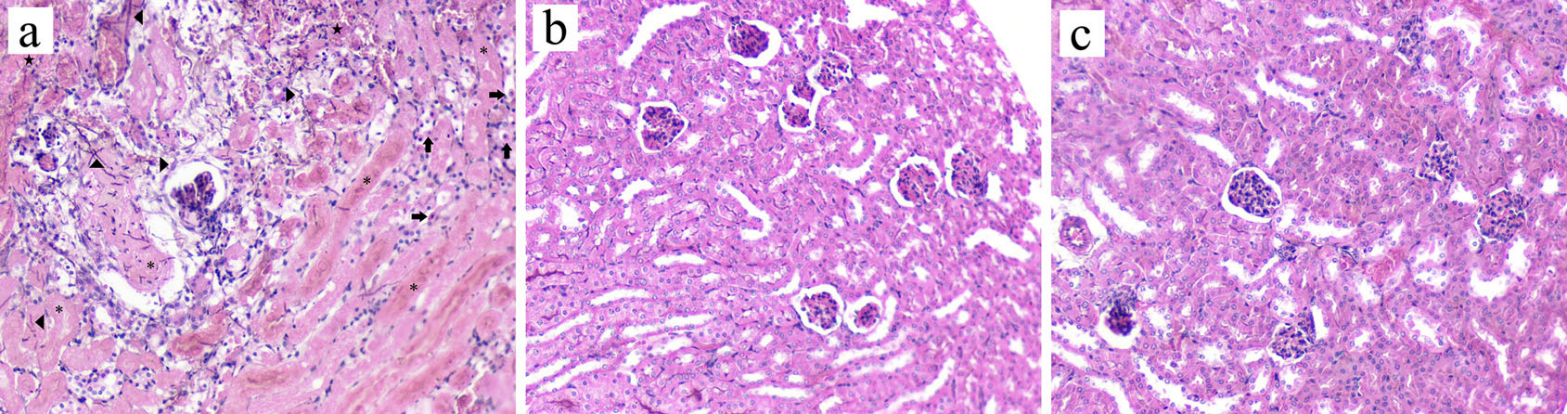
Histological findings in the kidney of immunocompromised BALB/c mice inoculated with *Aspergillus fumigatus* after five days of systemic infection. **(A)** Control: mice treated with placebo; **(B)** voriconazole: mice treated with 10 mg.kg^−1^ (572.5 µmoles per mouse); **(C)** ebselen: mice treated with 10 mg.kg^−1^ (765.8 µmoles per mouse) of ebselen. The treatments were performed intraperitoneally, twice a day, for four days. Tissues were stained with Grocott–Gomori’s methenamine silver (GMS) and hematoxylin and eosin (H&E); magnification, ×400. Asterisk: coagulative necrosis; arrow’s head: hyphae; arrow: mononuclear cell; star: hemorrhage.

Histopathological analyses showed a massive dissemination of hyphae in the kidneys from the control group (**Figure 2A**). Fungal hyphae were numerous and centered on the pelvis and secondarily extended to renal tubes of the medulla and cortex with presence of hyphae across the Bowman’s capsule and intact glomerulus. Additionally, the control group showed severe lesions with an extended area of coagulative necrosis and bleeding. Only in this group was there a diffuse inflammatory infiltrate with a predominance of mononuclear cells.

Concerning the groups treated either with voriconazole or ebselen (**Figures 2B, C**, respectively), in both, no fungal elements were detected on the entire kidney section observed. Kidney tissue was intact, and no evidence of inflammation was noted.

**Figure 3:**
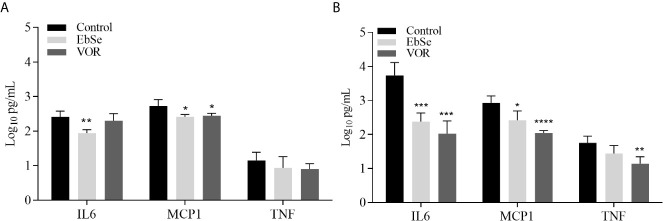
Systemic and local inflammatory cytokine evaluation in mice after treatment with EbSe or voriconazole. **(A)** Cytokines recovered from serum and **(B)** kidney homogenates from mice treated with placebo, EbSe, or voriconazole (VOR). *p < 0.05, **p < 0.01, ***p <0.001; ****p <0.0001, significant difference compared with control from each cytokine. TNF, tumor necrosis factor; MCP-1, macrophage/monocyte chemoattractant protein-1; IL-6, interleukin-6. Error bars correspond to the standard deviation.

Systemic cytokines (retrieved from the serum) (**Figure 3A**) and local cytokines (retrieved from kidney homogenates) (**Figure 3B**) showed similar expression patterns. The ebselen treatment was able to downregulate the expression of proinflammatory cytokine IL-6 and MCP-1 in both systemic and local responses (*p* < 0.05) (**Figure 3**). In the systemic response, only EbSe showed a significantly reduced expression of IL-6 (*p* = 0.0073) and MCP-1 (*p* = 0.0377). In this situation, voriconazole modulated only MCP-1 (*p* = 0.0132). In contrast, EbSe exhibited its greater effect on lowering the production of IL-6 (*p* = 0.0004) and MCP-1 (*p* = 0.0249) in kidney homogenates, although at a lower extent than in mice treated with voriconazole (IL-6, *p* = 0.0003; MCP-1, *p* < 0.0001; TNF, *p* = 0.0057) (**Figure 3B**).

## Discussion

Aspergillosis remains one of the main causes of death by invasive fungal infections (Lestrade et al., 2019). The incidence of azole-resistant strains has increased, mainly associated with the acquisition of resistant environmental strains which challenges the limited antifungal arsenal available (Cho et al., 2019; Lestrade et al., 2019). Therefore, the search for new treatment against aspergillosis is essential, and the drug repositioning tools have accelerated this process. Recently, Binder et al. (2020) showed that the TrxR protein is encoded by an essential gene for *A. fumigatus*, the *trxR* gene. Suppression of the *trxR* gene causes growth deficiency that is not supplied by supplementation of glutathione or other organic sources of sulfur, as occurs in yeasts. In addition, Marshall et al. (2019) elucidated the crystal structure of *A. fumigatus* thioredoxin reductase (AfTrxR) and described that the main mechanism of action of EbSe over *A. fumigatus* is the inhibition of AfTrxR. However, only *in vitro* studies were performed.

Our research group has been exploring the thioredoxin system as a promising drug target, with the selection of promising molecules for other pathogenic fungi (Capoci et al., 2019; Rodrigues-Vendramini et al., 2019; Faria et al., 2020). In this search for new mechanisms of action, different from those that are currently available, ebselen fitted our proposal well. Marshall and colleagues described that EbSe binds to Cys148 in the active site of thioredoxin reductase from *A. fumigatus*, locking AfTrxR in a catalytically nonproductive conformation (Marshall et al., 2019). This target of inhibition is totally different from those addressed in the commercial antifungal treatment, highlighting the possibility of EbSe in the treatment of refractory strains alongside the commonly used antifungals with usual targets (e.g., ergosterol). In addition, the selective manner in which EbSe links to human and fungi/prokaryotes TrxR confers TrxR as an excellent drug target (Ren et al., 2018).

EbSe has already been approved in a phase I clinical trial, in which safety, pharmacokinetic profile, and oral bioavailability in healthy humans were tested (Kil et al., 2017). In addition, this promising drug overcomes the hematoencephalic barrier acting in the central nervous system (Singh et al., 2016), an interesting feature for antimicrobial agents. Another clinical trial for the prevention of noise-induced hearing loss and treatment of mania or hypomania showed that doses of up to 600 mg twice daily did not change the hematological, serum chemistry, or radiological assessments between EbSe treatment and placebo groups also showing EbSe to be effective in the proposed treatment (Singh et al., 2013; Sharpley et al., 2020). Previous study of this group used 10 mg.kg^−1^ i.p. of EbSe to show its efficacy in the treatment against bipolar disorder (Singh et al., 2013). In an attempt to reproduce good results with safety and well tolerability in future human use, we treated mice infected with *A. fumigatus* by using 10 mg.kg^−1^ i.p. twice daily which allowed for a significant reduction of fungal burden. Just one *in vivo* study demonstrating the antimicrobial activity of ebselen using a model of *Caenorhabditis elegans* infection is described in the literature. The results showed that EbSe was more effective in reducing the fungal load of *Candida* and *Cryptococcus* over conventional antifungals such as amphotericin, fluconazole, and flucytosine (Thangamani et al., 2017). So far, there is no murine model showing antimicrobial EbSe efficacy.

In this study, a murine model allowed us to verify certain important points related to the host’s response to infection and treatment with EbSe, especially with histopathological and cytokine analyses. The treatments were shown to be efficient in reducing the fungal burden without exacerbating immune response which could be explained by the fast killing kinetics of EbSe as it was previously shown *in vitro* for *Candida* and *Cryptococcus* (Thangamani et al., 2017), which could also prevent the emergence of kidney lesions in the treated groups.

The decrease of proinflammatory cytokines could be associated with a reduction of infection and absence of hyphae, once the marked release of IL-6 occurs due to the exposition of hyphal fragments of *A. fumigatus* (Øya et al., 2019) TNF plays an important role in host immune defense against invasive fungal infections (Filler et al., 2005). In mice, the amount of TNF increases after 24 h, the acute phase response, and is associated with accumulation of large numbers of leukocytes at the foci of infection (Herbst et al., 2013). In this sense, the decrease of TNF levels could be correlated with kidney clearance and corroborated with histopathological analysis results.

Although the IV route does not mimic the natural route of infection in humans and involves organs that are not usually affected, such as the kidneys, this methodology provides greater accuracy and reproducibility of results, especially with assertive fungal inoculum for a reduced group of animals (Desoubeaux and Cray, 2017; Desoubeaux and Cray, 2018). In addition, this systemic proposed treatment can be extrapolated to a situation of invasive and systemic aspergillosis in antifungal EbSe activity evaluation. Thus, this study brings for the first time *in vivo* evidence of EbSe efficacy for invasive aspergillosis treatment, especially with a reduction of fungal burden. As a repurposing drug candidate, EbSe showed similar antifungal efficacy to conventional drugs, with a good safety profile and effectiveness. However, more animal studies are warranted in order to evaluate the potential role of EbSe as an alternative option for management of disseminated aspergillosis in humans.

## Data Availability Statement

The raw data supporting the conclusions of this article will be made available by the authors, without undue reservation.

## Ethics Statement

The animal study was reviewed and approved by the Institutional Ethics Committee for animal experimentation of the State University of Maringá, Brazil (Approval n° CEUA 9067030518). Collection and storage of *Aspergillus* clinical isolates were authorized by the Ethics Committee on Human Research of the State University of Maringá (Approval n° 2.748.843).

## Author Contributions

KS, IC, PC, FR-V, DF, and GA contributed to conception and design of the study. KS, PB-M and EK organized the database. PB-M and EK performed the statistical analysis. KS wrote the first draft of the manuscript. All authors contributed to the article and approved the submitted version.

## Funding

The authors acknowledge the Brazilian funding agencies CAPES (Coordenação de Aperfeiçoamento de Pessoal de Nível Superior), CNPq (Conselho Nacional de Desenvolvimento Científico e Tecnológico), and the State University of Maringa.

## Conflict of Interest

The authors declare that the research was conducted in the absence of any commercial or financial relationships that could be construed as a potential conflict of interest.
